# Extracellular Vesicle Transplantation Is Beneficial for Acute Kidney Injury

**DOI:** 10.3390/cells13161335

**Published:** 2024-08-12

**Authors:** Amankeldi A. Salybekov, Shigeaki Okamura, Takayasu Ohtake, Sumi Hidaka, Takayuki Asahara, Shuzo Kobayashi

**Affiliations:** 1 Kidney Disease and Transplant Center, Shonan Kamakura General Hospital, 1-1370 Okamoto, Kamakura 2478533, Japan; ohtake@shonankamakura.or.jp (T.O.); s_hidaka@shonankamakura.or.jp (S.H.); shuzo@shonankamakura.or.jp (S.K.); 2 Shonan Research Institute of Innovative Medicine, Shonan Kamakura General Hospital, 1-1370 Okamoto, Kamakura 2478533, Japan; okamura-shigeaki@nipro.co.jp (S.O.); t_asahara@shonankamakura.or.jp (T.A.); 3 Division of Regenerative Medicine, Department of Center for Clinical and Translational Science, Shonan Kamakura General Hospital, Okamoto 1-1370, Kamakura 2478533, Japan

**Keywords:** extracellular vesicles, renal ischemia-reperfusion injury, miR, regeneration-associated cells, angiogenesis

## Abstract

Under vasculogenic conditioning, certain pro-inflammatory subsets within peripheral blood mononuclear cells (PBMCs) undergo phenotypic transformation into pro-regenerative types, such as vasculogenic endothelial progenitor cells, M2 macrophages, and regulatory T cells. These transformed cells are collectively termed regeneration-associated cells (RACs). In this study, we aimed to investigate the therapeutic efficacy of RAC-derived extracellular vesicles (RACev) compared with a vehicle-treated group in the context of renal ischemia-reperfusion injury (R-IRI). Human PBMCs were cultured with defined growth factor cocktails for seven days to harvest RACs. EV quantity and size were characterized by nanoparticle tracking analysis. Notably, the systemic injection of RACev significantly decreased serum creatinine and blood urine nitrogen at day three compared to the control group. Histologically, the treatment group showed less fibrosis in the cortex and medullary areas (*p* < 0.04 and *p* < 0.01) compared to the control group. The CD31 staining confirmed enhanced capillary densities in the treatment group compared to the control group (*p* < 0.003). These beneficial effects were accompanied by angiogenesis, anti-fibrosis, anti-inflammation, and anti-apoptosis RACev miR delivery to ischemic injury to control inflammatory, endothelial mesenchymal transition, and hypoxia pathways. In vivo bioluminescence analysis demonstrated a preferential accumulation of RACev in the IR-injured kidney. The systemic transplantation of RACev beneficially restored kidney function by protecting from tissue fibrosis and through anti-inflammation, angiogenesis, and anti-apoptosis miR delivery to the ischemic tissue.

## 1. Introduction

Acute kidney injury (AKI) is a significant burden for healthcare systems, and its prevalence is estimated at about 20–200 cases per million people; among them, 7–18% of patients require hospital treatment and approximately 50% of patients are admitted to the intensive care unit (ICU) [1,2]. Importantly, AKI is associated with morbidity and mortality, and accounts for an estimated 2 million deaths worldwide every year, whereas AKI survivors are at high risk of developing chronic kidney disease (CKD) and end-stage renal disease (ESRD)—conditions that carry a high economic, societal, and personal burden [3]. Enhancing AKI treatment could significantly improve patient outcomes in hospitals. Despite numerous clinical trials exploring potential interventions, there remains a lack of reliable and specific treatments to effectively reverse AKI. Current management primarily focuses on optimizing hemodynamics, minimizing exposure to nephrotoxic substances, and utilizing renal replacement therapy [4]. Therefore, it is imperative to continue investigating new methodologies to address this condition.

Recent preclinical studies’ meta-analyses have highlighted that the transplantation of extracellular vehicles (EVs) can significantly improve AKI [5]. Hence, EV therapy may be considered a promising treatment option to cure AKI. EVs are a heterogeneous population of membrane-bound vesicles that are central messengers for intercellular communication [6]. EVs can be divided into three group depending on the size range, small (<100 nm), medium (<200 nm), or large (>200 nm), and usually express tetraspanin family CD63+, CD9+, CD81+, Annexin A5, etc., surface markers [7]. They are released by all of the cell types and can be classified into two main categories depending on their origin. Small EVs (previously known as exosomes) are released by the endosomal compartment and have an average size of 100 nm, and medium or large EVs are released by the budding of the plasma membrane and range in size from microvesicles to large vesicles (50 nm to 1 µm) [7].

The vasculogenic conditioning of peripheral blood mononuclear cells (PBMCs) enriches M2 macrophages, vasculogenic EPCs (also known as definitive EPCs), and reparative regulatory T and B cells, also known as regeneration-associated cells (RACs). The therapeutic efficacy of RACs has been shown in cardiovascular diseases [8]; however, the therapeutic effect of RACev on AKI has not been investigated yet. In this study, we aimed to investigate the therapeutic efficacy of RAC-derived extracellular vesicles (RACev) in the context of renal ischemia-reperfusion injury (R-IRI).

## 2. Materials and Methods

### 2.1. Cell Culture

PBMCs were isolated from healthy human participants after receiving written informed consent according to the institutional ethical review board (CS-00201623). In a brief procedure, 30 mL of peripheral blood was collected using a BD vacutainer (Frankline Lakes, NJ, USA). The cells were then cultured in a growth-factor-enriched medium, specifically Stem Line II (Sigma-Aldrich, St. Louis, MO, USA), supplemented with 100 ng/mL recombinant human stem cell factor (rhSCF), 100 ng/mL Flt-3 ligand (rhFl3Lig), 20 ng/mL thrombopoietin (rhTPO), 50 ng/mL vascular endothelial growth factor (rhVEGF), and 20 ng/mL interleukin-6 (rhIL-6), all sourced from Peprotech, Inc. (Rocky Hill, NJ, USA). Additionally, penicillin/streptomycin (100 U/100 μg/mL) from Gibco was added to the culture. Peripheral blood mononuclear cells (PBMCs) were cultured at a concentration of 1 × 10^7^ cells per 10 cm dish (Sumitomo Bakelite Co., Tokyo, Japan) and incubated at 37 °C in a humidified atmosphere containing 5% CO_2_, following previously established protocols [8,9].

### 2.2. Cell Phenotype Characterization

PBMCs and cultured regeneration-associated cell surface antigens such as CD34 (clone 581), CD133 (clone AC133), CD11b (clone M1/70), CD206 (clone 15-2), CD11c (clone S-HCL-3), CD3 (clone UCHT1), CD4 (clone RPA-T4), CD8 (clone SK1), CD25 (clone BC96) and CD127 (clone A019D5) and PI (all from Biolegend Ltd., San Diego, CA, USA) were analyzed by flow cytometry to determine their positiveness, as reported elsewhere. Briefly, immediately after cell suspension in FACS buffer at 4 × 10^5^ cells/200 μL, 1 μL of FcR blocking (Miltenyi Biotec., Gladbach, Germany) was added to reduce unspecified binding and it was left at 4 °C for 20 min. Subsequently, the antibodies, as mentioned above, were added according to the manufacturer’s manual and left at 4 °C for 30 min. Stained cells were washed twice, and flow cytometric analysis was performed on a BD FACS Verse and Fortessa (BD, Franklin Lakes, NJ, USA). The raw data were analyzed using FlowJo (BD 10.6 version, Franklin Lakes, NJ, USA) and FCS Express version 6.2. (Denova Software Ltd., Pasadena, CA, USA)

### 2.3. Endothelial Progenitor Cell Colony Formation Assay

Freshly isolated PBMCs or RACs were seeded at 1.5 × 10^5^ cells into a semisolid methyl cellulose-based culture medium-coated 35 mm dish (BD Falcon, CA, USA) and left in a humidified incubator with 5% CO_2_ at 37 °C until EPC colony appearance. The number of adherent colonies on the dishes were counted between days 16 and 18 using a gridded scoring dish (STEMCELL Tech Inc., Vancouver, BC, Canada) under a phase-contrast light microscope (Eclipse TE3000; Nikon, Tokyo, Japan). Primitive EPC colony-forming units (PEPC-CFUs) and definitive EPC colony-forming units (DEPC-CFUs) were counted separately, as reported elsewhere [10,11].

### 2.4. Extracellular Vesicle Isolation and Characterizations

Following the collection of RACs, the cells were cultured in X-vivo 15 media (Lonza, Morristown, NJ, USA) enhanced with 5% exosome-depleted fetal bovine serum (FBS) from Gibco for 48 h. The CCM was then carefully decanted into a clean tube, and cellular debris was eliminated through sequential centrifugation at 300× *g* for 10 min at 4 °C and 2000× *g* for 20 min at 12 °C. Subsequently, the CCM was filtered through a 0.2 um filter (Millipore Merck, Burlington, MA, USA) to exclude particles larger than 200 nm. This filtered CCM was then transferred to new ultracentrifuge tubes (Ultra clear, cat# 344059, rotor type Ti40SW, Beckman Coulter, Brea, CA, USA) and ultracentrifuged at 174,000× *g* for 110 min at 4 °C to obtain a pellet. The quantity and size of the EVs were analyzed using nanoparticle tracking analysis with a NanoSight 500^®^ (Malvern Pananalytical Co., Ltd., Malvern, UK), following the manufacturer’s protocols. The structure of the EVs was verified using standard transmission electron microscopy (JEM-1400, Jeol Co., Ltd., Tokyo, Japan).

### 2.5. EV Labeling and Tracking

After passing the CCM through a 0.2 μm filter (Millipore Merck), we added 2 μM of CM-DiI dye (C7000, Thermo Fisher, Waltham, MA, USA) to the CCM and allowed it to incubate for 30 min to stain the EVs. Subsequently, this stained CCM was transferred to an ultracentrifuge tube and spun at 174,000× *g* for 110 min at 4 °C. The stained EVs accumulated at the tube’s bottom and were then washed twice with 1× phosphate-buffered saline (PBS), following previously described methods [12]. The EV-enriched pellet was resuspended in a minimal volume (30–50 μL) of a suitable buffer, depending on the subsequent experiments, following EV isolation. Initially, the labeled RACev and a control saline solution were administered intravenously via the tail using a 24 G angiocatheter (Terumo, Tokyo, Japan) on the first day following the start of R-IRI. Three hours after administering the EVs, the animals were euthanized. Their organs, including the kidneys, spleen, lungs, and heart, were collected for further analysis of EV distribution, as previously described [13]. To assess EV localization at the injury site, the IVIS Lumina III system (Perkin Elmer, Waltham, MA, USA) was employed. The excitation and emission filters for DiI dye were adjusted to 520 nm and 570 nm, respectively [13].

### 2.6. Flow Cytometry Analysis of EVs

All experiments to measure EV sizes using flow cytometry were conducted using a 13-color, 3-laser Beckman Coulter DxFlex Flow Cytometer, which included lasers at wavelengths of 405 nm, 488 nm, and 638 nm (Beckman Coulter, Brea, CA, USA). The system’s configuration was specifically adapted to enhance the detection of small EVs. Specifically, the 405/10 VSSC filter was moved to the V450 channel in the wavelength division multiplexer (WDM), and the detector’s settings were updated using CytExpert software version 2.5 to assign the VSSC channel within the WDM. Cleaning procedures, including the use of FlowClean and water filtered through a 10 μm filter, were implemented before acquiring data to minimize debris and background interference. For EV characterization, fluorophore-labeled antibodies targeting CD9 (PE, #312106), CD63 (PE, #353004), Alix (Alexa 594, #634504), and Hsp-70 (Alexa 488, #648003) were used (all sourced from BioLegend, CA, USA). Additionally, latex beads with diameters of 100 nm and 200 nm (from Beckman Coulter) served as standards to assess EVs and to calibrate the flow cytometry acquisition settings.

### 2.7. Animals

All procedures involving animals were conducted at Shonan Health Innovation Park in compliance with the standards set by AAALAC International (approval # AU-00030960). Male Lewis rats, with body weights ranging from 150 to 250 g, were acquired from Charles River Laboratories (Yokohama, Japan) through Oriental Yeast Co., Ltd. (Tokyo, Japan). The animals were maintained under standardized environmental conditions: a temperature of 20 ± 2 °C, relative humidity between 50 and 60%, and a 12 h light/dark cycle. They had continuous access to water and food.

### 2.8. Renal Ischemia-Reperfusion Injury Induction

The rats were sedated using 3–4% sevoflurane (Maruishi Pharmaceutical Co., Ltd., Tokyo, Japan). Once adequately anesthetized, a midline incision was made in the abdomen, and the renal pedicles were exposed and clamped for 45 min using microaneurysm clamps (F.S.T. Esssen, Essen, Germany). To maintain body temperature during the ischemic period, the rats were placed on a heating pad set to 37 °C. After the clamps were removed, the kidneys were examined to ensure the restoration of blood flow and the return to their natural color before suturing the abdominal incision. Rats in the sham operation group underwent the same surgical steps without the application of the clamps. On the day following the induction of R-IRI, transplantation procedures were carried out. The treatment group received extracellular vesicles derived from 1 × 10^6^ regeneration-associated cells, while the control group received PBS. Both were administered intravenously through the tail vein using a 24 G angiocatheter (Terumo, Japan), as previously described [8]. After the end of each experiment, the animals were euthanized using a lethal dose of 5% sevoflurane. Once euthanasia was confirmed, essential organs including the kidneys, lungs, spleen, and liver were collected.

### 2.9. Immunohistochemistry Analysis

The kidneys were perfused with heparinized phosphate-buffered saline (PBS, 1000 U/500 mL), and then fixed in 4% paraformaldehyde (Cat #163-20145, Fujifilm Co., Ltd., Tokyo, Japan) overnight. Subsequently, they were embedded in paraffin and sectioned into slices ranging from 2.5 to 3 μm in thickness. The sections were stained with Periodic Acid-Schiff (PAS, on days 4 and 28 after the onset of R-IRI) and Masson Trichrome (on day 28) to determine the size of the fibrosis, as previously described [8]. To analyze microvascular density, the sections were incubated with anti-CD31 (1:100) antibody and DAB [8]. All histology photographs were taken under a microscope at ×200 magnification using an all-in-one fluorescence microscope BZ-X800–Keyence. (Keyence Co., Ltd., Tokyo, Japan). The histology data were measured from 3 to 5 high-power fields per section of cortical and medullary areas using Keyence Analyzer (Keyence Co., Ltd., Tokyo, Japan).

### 2.10. Library Preparation, Sequencing, and Bioinformatics Analysis

The CCM-derived small and large RNAs were fractionated using the miRNeasy mini kit (Cat#217084, Qiagen, Germantown, MD, USA) and the RNeasy MinElute Cleanup Kit (Cat#74204, Qiagen, USA). Sequencing libraries were constructed according to the manufacturer’s protocols using the QIAseq™ miRNA Library Kit (Cat#331505, Qiagen, Hilden, Germany). Library quality was assessed with an Agilent Bioanalyzer using a high sensitivity DNA chip (Agilent Technologies, Santa Clara, CA, USA). The pooled libraries were sequenced using NextSeq 500 (Illumina, Inc., San Diego, CA, USA) in 76-base-pair (bp) single-end reads. The QIAseq miRNA library kit adopts a unique molecular index (UMI) system, enabling the unbiased and accurate quantification of mature miRs. Original FASTQ files generated using NextSeq were uploaded to the Qiagen GeneGlobe Data Analysis Center (https://geneglobe.qiagen.com (accessed on 12 March 2024)) and aligned to miRBase v21 (http://www.mirbase.org (accessed on 12 March 2024)). All reads assigned to particular miRs were counted, and the associated UMIs were aggregated to count unique molecules. A matrix of the miR UMI counts was subjected to downstream analyses using StrandNGS 3.4 software (Agilent Technologies, Santa Clara, CA, USA). The UMI counts were quantified using the trimmed mean M-value (TMM) method [14]. To determine the target genes of differentially expressed miRs, we integrated all the information on PITA miRBase version 18, microRNA.org miRBase version 18, and TargetScan version 6.0.

Kidney tissues were collected on day four after the onset; R-IRI and total RNA was isolated using the QuickGene Auto12S nucleic acid extraction system (Kurabo, Co., Ltd., Osaka, Japan), according to the manufacturer’s instructions. The RNA integrity score was confirmed by Agilent Bioanalyzer (Agilent Technologies, Santa Clara, CA, USA). The Affymetrix GeneChip Clarriom S (Cat # 902935 Affymetrix, Santa Clara, CA, USA) was used. Rat microarray was used, and the latter contains probes that cover more than 20,000 rat genes. The data were analyzed in Transcriptome Viewer software v1.

Next, to determine the biological function of the target genes, Gene Ontology (GO) analysis and pathway statistical analysis were performed. Pathway statistical analysis to determine the pathways considering the number of genes in the pathway and the number of target genes was performed on the pathway collection of the Wiki Pathways database [15] using the PathVisio tool [10].

### 2.11. Statistical Analysis

All data are presented as mean ± standard error of the mean (SEM). The Mann–Whitney U test was employed for analyzing two groups with non-parametric data. When comparing multiple groups across different time points, two-way ANOVA was utilized, followed by Tukey’s post hoc analysis for specific pairwise comparisons. MicroRNAs (miRs) showing a fold change of two or greater were identified as differentially expressed. All statistical procedures were conducted using GraphPad Prism version 9.1 (GraphPad Prism Software Inc., San Diego, CA, USA), and a *p*-value of less than 0.05 was considered statistically significant.

## 3. Results

### 3.1. Characterization of Regeneration-Associated Cells

**Figure 1:**
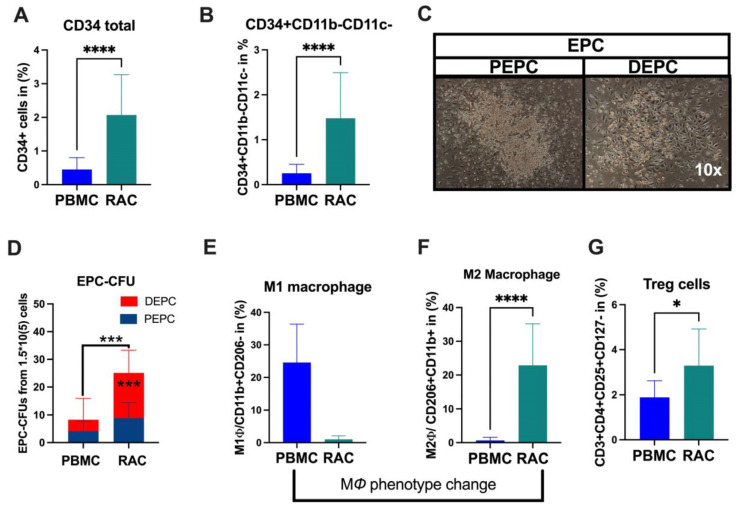
**Characterization of RACs.** (**A**) Total stem and progenitor levels increased after vasculogeneic conditioning. (**B**) The EPCs were quantitatively and (**C**,**D**) qualitatively enhanced in post-vasculogenic culture (the majority by definitive EPC expansion). (**E**,**F**) Vasculogenic conditioning dramatically accelerated M1 macrophage phenotype conversion to regenerative macrophage type 2 (**G**). The level of regulatory T cells. * *p* < 0.05; *** *p* < 0.01; **** *p* < 0.0001 vs. the control group; Statistical significance was determined using a Mann–Whitney test. n = 10 per group. The results are presented as mean ± SEM. * *p* < 0.05; *** *p* < 0.01; **** *p* < 0.0001.

Under vasculogeneic conditioning, the total CD34+ cell and EPC (CD34+CD11b-CD11c-) levels statistically significantly increased RAC in comparison with PBMC (Figure 1A, CD34+PBMC vs. CD34+RAC, 0.48 vs. 1.6%, *p* < 0.0001; Figure 1B, EPC-PBMC vs. EPC-RAC, 0.19 vs. 1.13%, *p* < 0.0001). Then, we evaluated colony-formation capability using EPC-CFA analysis (Figure 1C). The results demonstrated that RAC’s total and definitive EPC or vasculogeneic EPC colonies markedly increased, indicating qualitative and quantitative EPC improvement (PBMC vs. RAC, 4.1 vs. 15, *p* < 0.0001) (Figure 1C,D). The pro-inflammatory cell phenotype dynamically changed to an anti-inflammatory phenotype cell subset under vasculogeneic culture conditioning. In particular, vasculogenic conditioning dramatically accelerated M1 macrophage phenotype conversion to regenerative macrophage type 2 (M1ϕ-PBMC vs. M2ϕ-RAC, *p* < 0.001) (Figure 1E,F). These data indicate that the vasculogenic conditioning of PBMNCs enriched M2ϕ by converting the classical M1ϕ phenotypes into the alternative activated M2ϕ phenotype (Figure 1E,F). The regulatory T cells significantly increased in RAC (PBMC-1.7 vs. RAC-2.8, *p* < 0.04) (Figure 1G). To sum up, vasculogenic conditioning enhanced pro-regenerative cell subsets such as definitive EPC, M2ϕ, and regulatory T cells, collectively named regeneration-associated cells (RACs).

### 3.2. Characterization of EVs

**Figure 2:**
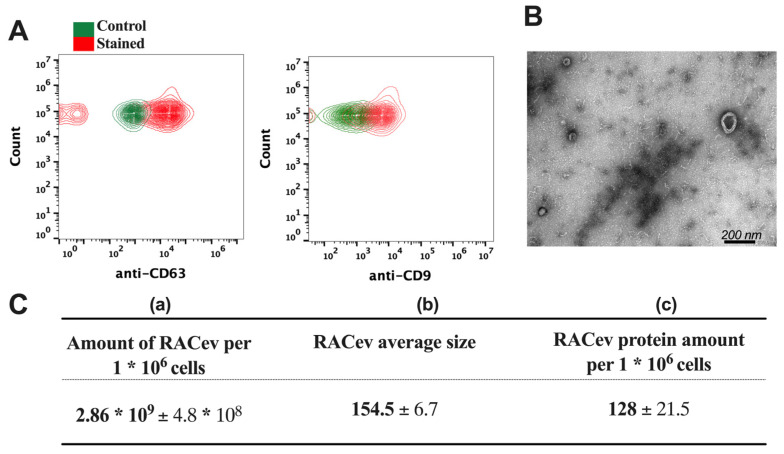
**Characterization of RAC-derived extracellular vesicles.** (**A**) EV-specific anti-CD63 and anti-CD9 biomarker expression in RACev. (**B**) Representative transmission-electron microscopy figures showed the lipid bilayer structure in RACev. (**C**) (**a**) Quantification of one million RAC-derived EVs, (**b**) average size, and (**c**) protein amount.

The flow cytometry analysis confirmed the expression of EV-specific markers such as CD9 and CD63 in RACev (Figure 2A). Transmission electron microscopy (TEM) revealed a bi-layered membrane structure (Figure 2B). The average number of EVs derived from one million RACs measured by a nanoparticle tracking analysis machine (Figure 2C(a,b)) was 2.86 × 10^9^ ± 4.8 × 10^8^ and the average size was 154.5 ± 6.7 nm, respectively. Then, we quantified the total protein amount of RACev derived from one million RACs using a BSA assay (Figure 2C(c)). Altogether, the isolated RACev expressed EV-specific markers, and their size range and TEM findings complied with the MISEV 2018 guidelines [7].

### 3.3. RACev Transplantation Improved Kidney Function

**Figure 3:**
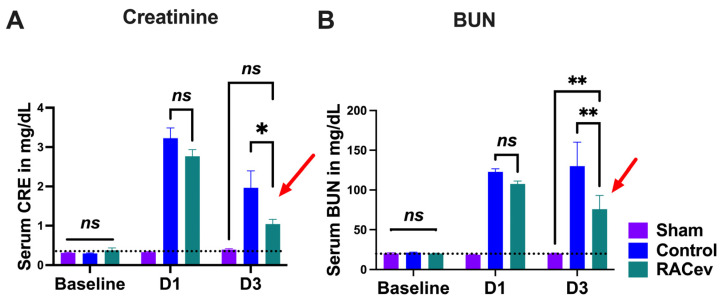
**RACev transplantation restored kidney function.** (**A**) Serum creatinine level at day three significantly decreased in RACev vs. control. (**B**) Similarly, serum BUN level was dramatically diminished in the RACev transplanted group compared to the Control group. * *p* < 0.05; ** *p* < 0.01; ns is not significant vs. the control group; statistical significance was determined using a 2-way ANOVA followed by Tukey’s multiple comparison test. The results are presented as mean ± SEM.

The rat body weight (BW) was measured before and every week after onset of R-IRI. At four weeks, BW in the RACev transplanted group significantly increased in comparison with the control (RACev; 333 ± 6 g vs. control; 315 ± 5.5 g, *p* < 0.0053) counterparts (Figure S1A). We adjusted the treatment time on day one after the onset of R-IRI with a dose of 1 × 10^6^ cells and derived EV injection via the tail vein based on a clinical treatment regime. Serum Creatinine (sCr) significantly decreased at day three after the onset of R-IRI in the EV-transplanted group compared to the control groups (*p* < 0.019, RACev vs. control) (Figure 3A). The serum blood urea nitrogen (BUN) level was diminished markedly in the RACev group compared to the control groups (*p* < 0.005, RACev vs. control) (Figure 3B). To sum up, RACev transplantation beneficially improved kidney function compared to the control group.

### 3.4. RACev Therapy Preserved from Kidney Fibrosis

**Figure 4:**
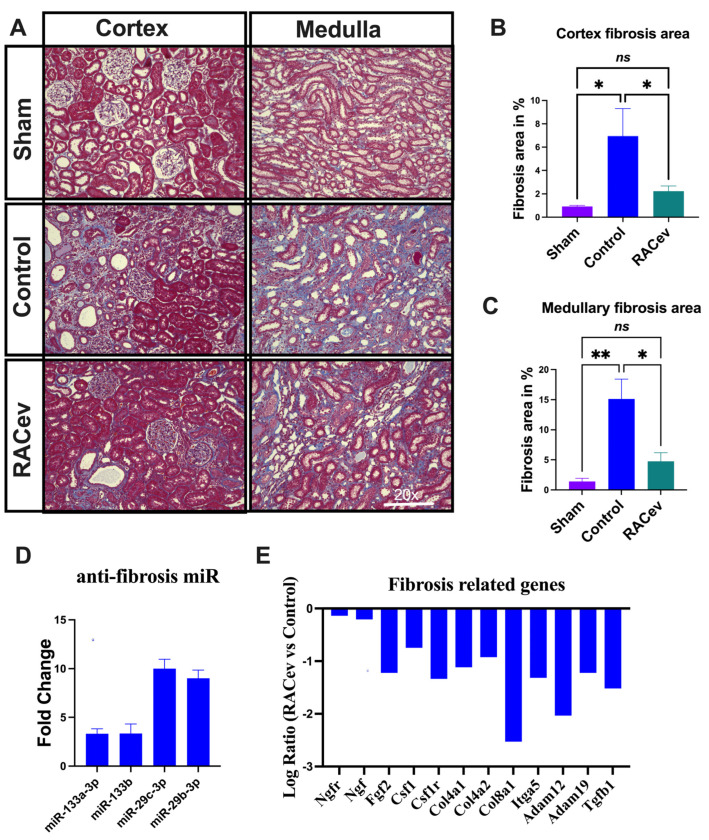
**RACev transplantation preserved renal interstitial fibrosis.** (**A**) Representative Masson trichrome staining depicts reduced or preserved fibrosis area in RACev-transplanted group in comparison to control groups. (**B**) Fibrosis area quantification in cortex area and (**C**) medullary area. (**D**) Anti-fibrosis miRs were significantly upregulated in RACev. (**E**) Fibrosis-related genes markedly upregulated RACev vs. control four days after the onset of R-IRI. * *p* < 0.05; ** *p* < 0.01; ns is not significant vs. the control group. Transcriptome analysis at day four after the onset of AKI demonstrated fibrosis-related gene upregulation in control group vs. RACev. Statistical significance was determined using a one-way ANOVA followed by Dunn’s multiple comparison test. The results are presented as mean ± SEM (n = 8–10 per group).

Masson trichrome staining depicted a reduced kidney fibrosis level in the RACev treatment group compared to the control group at four weeks in either the cortex or medullary area (*p* < 0.04, RACev vs. control of the cortex area; *p* < 0.01, RACev vs. control of the medullary area), respectively (Figure 4A–C). Mechanistically, this anti-fibrotic effect of RACev may be coupled with anti-fibrosis miR’s delivery to the ischemic tissue (the latter was abundantly expressed in RACev), which was beneficially preserved from excessive inflammation and scar formation (Figure 4D). Furthermore, kidney tissue gene expression analysis revealed that the majority of pro-fibrotic genes (NGFR, FGF2, CSFLR, COL4A1, COL8A1, TGFb1, etc.) markedly downregulated at day four after the onset of R-IRI in the RACev-treated group compared with the control group (Figure 4E). Together, RACev transplantation significantly reduced kidney fibrosis accumulation via anti-fibrosis-related miR delivery.

### 3.5. Regulatory Role of RACev on Epithelial Mesenchymal Transition, Inflammation, and Hypoxia after Ischemic Injury

**Figure 5:**
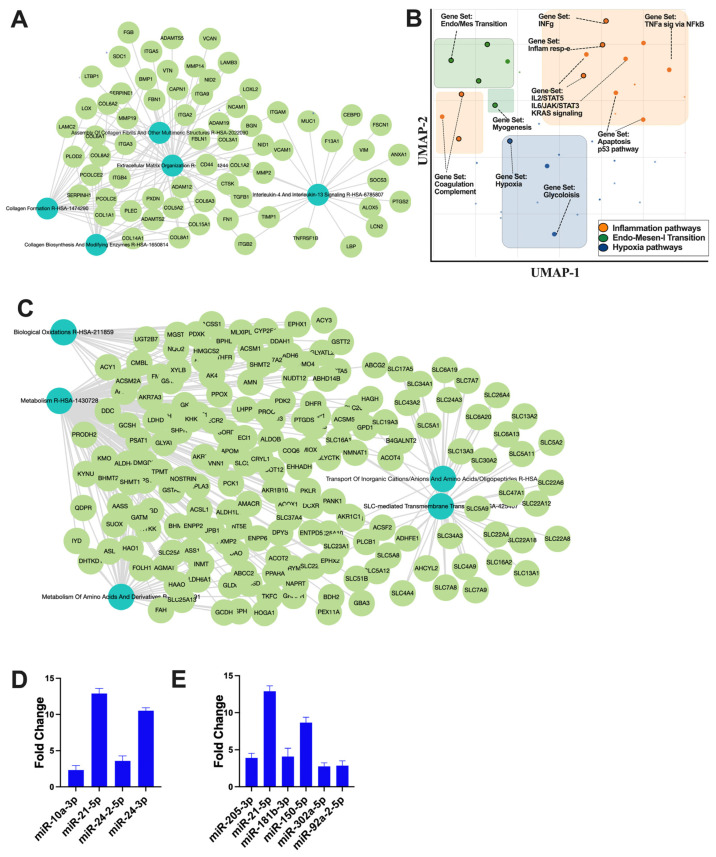
**Regulation of inflammatory and apoptosis pathways.** (**A**) Gene co-expression network analysis of control group revealed (**B**) inflammation, EMT, and hypoxia pathway upregulation. (**C**) RACev-transplanted group demonstrated regeneration-associated pathway upregulation. (**D**) Anti-inflammatory miRs are abundantly expressed in RACev. (**E**) Anti-apoptotic and proliferation-associated miR expression in RACev. Differentially expressed miRs were determined using a threshold of absolute values of fold change ≥ 2.

The small non-coding RNA sequencing and data analysis revealed that RACev contains several crucial anti-inflammatory miRs such as miR-10a-3p, miR-21-5p, miR-24-2-5p, and miR24-3p, which regulate from excessive inflammation and initiate regeneration (Figure 5A). Moreover, RACev carries anti-apoptosis and cell proliferation miRs (Figure 5B). The gene ontology and pathway analysis revealed that kidney tissue fibrosis in control groups were initiated due to hypoxia pathway (*p* < 2.19 × 10^−2^) (Figure 5C,D) and excessive upregulation of inflammatory pathways such as IL-6-JAK2-STAT3 (*p* < 1.43 × 10^−3^), TNFa signaling via NF-kB (*p* < 1.51 × 10^−3^), and INFg response (*p* < 2.19 × 10^−2^). The latter initiated an early epithelial–mesenchymal transition pathway (*p* < 3.75 × 10^−11^) that was also confirmed by network analysis (Figure 5C,D). The gene co-expression network analysis of the RACev-treated group demonstrated an enrichment of metabolism, biological oxidation, and the metabolism of amino acids and derivatives (Figure 5E). The latter is important for ischemic injury restoration.

### 3.6. RACev Restored Capillary Densities

**Figure 6:**
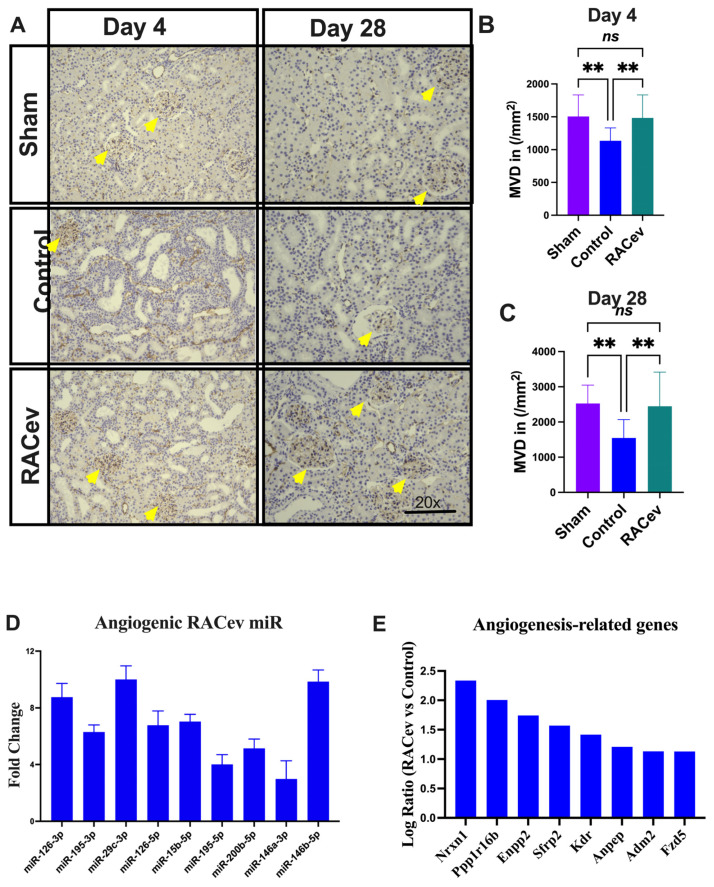
**Enhanced angiogenesis in infarcted tissues.** (**A**) Microvascular density was enhanced in ischemic injured kidney tissue in the RACev-transplanted group. (**B**) CD31 positive capillary count at day four and (**C**) day 28 after onset of R-IRI. (**D**) Angiogenic miRs, also known as angiomiRs, are markedly expressed in RACev. (**E**) Angiogenesis-related gene expression of RACev vs. control groups’ kidney tissues. ** *p* < 0.01; ns is not significant vs. control group. Statistical significance was determined using one-way ANOVA with Dunn’s multiple comparisons test. Results are presented as the mean ± SEM (n = 8–10 per group). Differentially expressed miRs were determined using a threshold of absolute values of fold change ≥ 2.

Capillary density was evaluated in the acute phase on day 4 (D4) and day 28 (D28) after the onset of R-IRI to verify its vascular tissue-protective (D4) and vasculogenic effects (D28). Representative Figure 6A–C show that the microvascular density of the ischemia-injured kidney tissues were beneficially enhanced in the RACev transplanted group compared to the control groups (*p* < 0.005, RACev vs. control at D4; and *p* < 0.003 at D28), indicating the strong valculoprotective and vasculogenic properties of RACev. Moreover, non-coding miR analysis revealed that the key pro-angiogenic miRs responsible for angiogenesis, so-called “angiomiRs” such as miR-126-3p/5p, miR146a-p, miR-200b-5b, etc., were highly upregulated in RACev (Figure 6D). Transcriptome analysis disclosed that the RACev therapy significantly upregulated vasculogenesis-associated genes in the ischemic kidney tissue at day four in comparison to the control group (Figure 6E). To sum up, RACev delivered vasculoprotective miRs and angiomiRs at the site of ischemic injury to protect from hypoxia-induced kidney vascular network damage and enhanced vasculogenesis.

### 3.7. Preferential RACev Accumulation in the Injured Kidney

**Figure 7:**
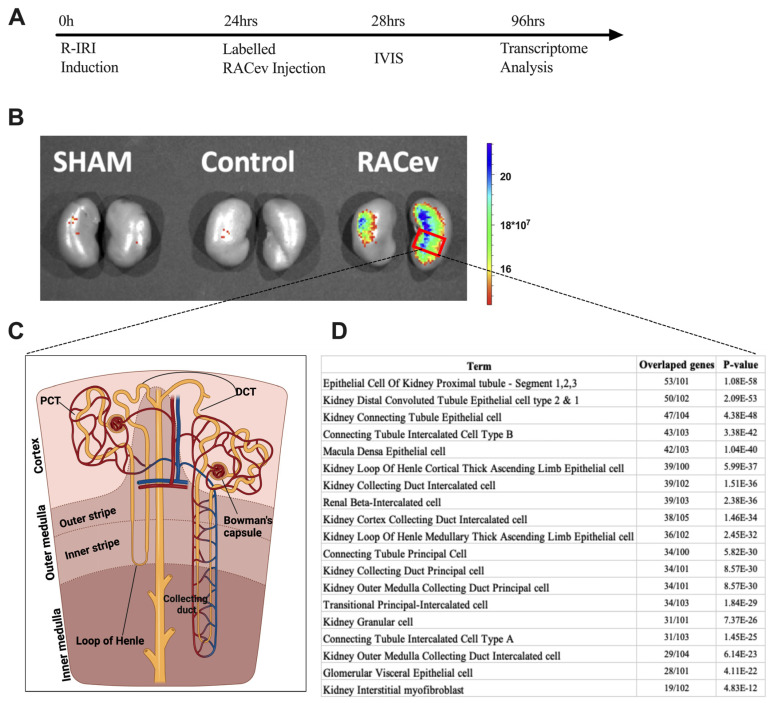
**Selective accumulation of RACev.** (**A**) Schematic design of an in vivo study. (**B**) Systemic transplantation of labeled RACev preferentially accumulated into the ischemia-injured kidneys. (**C**,**D**) Transcriptome cell annotation and mapping showed possible RACev accumulation in the kidney.

The IVIS analysis results demonstrated that the labeled RACev preferentially accumulated in the injured kidney (mostly the medullary area) (Figure 7A,B). Then, we employed cell type annotation analysis using the most upregulated 500 genes to map or identify which area of nephrons/cells are highly expressed in RACev vs. control. As shown in Figure 7D, the top 10 most upregulated nephron areas were proximal tubule epithelial cell segments one, two, and three, distal convoluted tubule cell types one and two, and kidney connecting tubule epithelial cells. The results prove an IVIS outcome where most of the labeled RACev accumulated between the cortex and medullary area. On the other hand, a small portion of the transplanted RACev were also trapped in the spleen. In conclusion, EV tracking and transcriptome analyses revealed that RACev selectively accumulated in ischemic renal tissues.

## 4. Discussion

In the present study, our results revealed that human regeneration-associated cell-derived EVs significantly improved kidney functional and morphological parameters in a rat R-IRI model. The first response to kidney ischemia injury is inflammation [11,16]. Ischemic injury leads to the loss of renal tissue architecture and the release of inflammatory mediators, which induce the accumulation of mononuclear cells in the renal interstitium to scavenge debris cells and initiate the reparatory phase [17]. In the present study, fibrosis was dramatically reduced in the RACev treatment group vs. the control group. Of note, fibrosis is the final end-point of any injury or damage. From this point of view, it is worst to discuss molecular mechanisms in early acute or subacute phases of AKI. Pathway and gene-co-expression network analysis revealed that the three main pathways driving kidney tissue fibrosis in control were inflammatory pathways, the hypoxia response pathway, and the early EMT pathway. Similarly, recent single-nucleus transcription analysis also revealed that the activation of NF-κB–, TNF-(all), and AP-1 signaling pathways plays a key role in kidney maladaptive repair [18]. Moreover, failed resolution can cause partial EMT, cell senescence, G2/M cell cycle arrest, the activation of fibroblasts and immune cells, and epigenetic changes that perpetuate inflammation and release a profibrogenic secretome. All of the above-mentioned factors contribute to the progressive decline of kidney function and fibrosis, also known as maladaptive repair [11,19]. Earlier studies demonstrated that stem and progenitor cells enhanced ischemic kidney injury recovery via various beneficial anti-inflammatory, anti-apoptotic, and anti-fibrosis miR delivery [5,20]. Similarly, RACevs carry anti-inflammatory, anti-fibrosis, and anti-apoptotic miR families such as miR-21, miR-10, miR-29, and miR-150, and all these miRs play essential roles in adaptive repair after the onset of kidney injury, as shown in earlier studies [21,22,23,24,25]. For instance, RACev abundantly expressed anti-fibrosis miR-29c-3p and miR-29b-3p, collectively known as the anti-fibrosis miR-29 family [21,22,26]. Compelling evidence demonstrates that an overexpression of miR-29b significantly reduces the *TGFb-1*-induced expression of collagens I and III by renal tubular cells in an experimental model [21,26]. A previous study revealed that miR-29b delivery via ultrasound, either before or after established obstructive nephropathy, prevented kidney fibrosis [26]. Notably, whole-transcriptome analysis revealed that RACev-transplanted group fibrosis-related genes are markedly downregulated, such as *TGFb-1* and collagen family genes, indicating that RACev delivers anti-fibrosis miR to the injured kidney to protect against fibrosis. Taken together, RACev delivers crucial anti-inflammatory, anti-fibrosis, and anti-apoptotic miRs for the effective protection and early initiation of regeneration processes after the onset of R-IRI.

In our study, we found that RACev therapy effectively protects vascular networks via beneficial angio-miR delivery in kidney injury. Interestingly, at D4 and D28 after the onset of R-IRI, the functional CD31+-stained capillary numbers were significantly higher in the RACev group compared to the control, indicating vasculoprotective effects in early injury periods (D4, protection from endothelial injury and loss) and neovasculogenesis later on (D28). In a previous study, Cantaluppi et al. [27] demonstrated a therapeutic effect of EPCevs in a rat AKI model. The authors reported that EPCevs increased cell proliferation and reduced apoptosis in tubular epithelial cells, along with vasculogenesis enhancement via transferring proangiogenic miRs such as miR-126 and miR-296. Similarly, our in silico analysis demonstrated that the angiomiRs are abundantly upregulated in RACev. The TargetScan analysis disclosed that the angiomiRs corresponded with predicted regions of vasculogenesis-related genes. Of note, miR-126 might exert dual effects depending on endothelial segmental regions, for instance, the heterogenic response of renal microvascular endothelial cells to inflammatory stimuli, as indicated earlier [28]. Recent single-cell sequencing highlighted 24 renal endothelial cell phenotypes (of which 8 were novel), and the complete heterogeneity of these cells between and within the cortex, glomeruli, and medulla [29]. Consequently, RACev carries various angiomiRs and the latter might be effective in diverse kidney endothelial cell recovery from ischemia injury and may boost the vasculogenesis process. The IVIS findings and 500 highly upregulated gene cell annotation and mapping analysis revealed that systemic transplanted RACev selectively accumulated at the ischemia-injured tissues. The RACev accumulated outer medulla outer and inner stripes for i.g proximal tubule epithelial cell segments I, II, and III, distal convoluted tubule cell types I and III, and kidney connecting tubule epithelial cell clusters. Collectively, these regions mentioned above are considered ischemia-vulnerable nephron segments, proving beneficial ensemble works of RACev delivery (such as angimiRs, anti-inflammation, anti-apoptotic, and anti-fibrosis effects) at the right time and in the right place.

The study’s limitations include (i) the use of xenogeneic transplantation (human RACev to rat R-IRI model); and adverse effects have not been evaluated. Prior to translation to the clinic, for the latter, the immunological response to RACev in larger animals must be more precisely evaluated, and the (ii) optimal dose of RACev should also be determined.

## 5. Conclusions

To conclude, RACev transplantation effectively restored AKI, induced by ischemia in a rat model via (i) anti-fibrosis, (ii) anti-inflammation, (iii) neovascularization-associated miRs, and (iv) preferential accumulation in the ischemic renal tissue.

## Data Availability

The original contributions presented in the study are included in the article/Supplementary Materials, further inquiries can be directed to the corresponding author.

## Supplementary Materials

The following supporting information can be downloaded at: https://www.mdpi.com/article/10.3390/cells13161335/s1. Figure S1: Animal body and organs weight at 4 weeks.
