# Supplementary material for: Extracellular Vesicle Transplantation Is Beneficial for Acute Kidney Injury
Source: Cells. 2024 Aug 12;13(16):1335. doi: 10.3390/cells13161335 (PMC11352623; doi:10.3390/cells13161335)
Supplement: Supplementary file 1 [file cells-13-01335-s001.zip › cells-3132272-supplementary.pdf]

Extracellular Vesicles Transplantation Is Beneficial For Acute Kidney Injury

Amankeldi A. Salybekov[1,2*], Shigeaki Okamura[2], Takayasu Ohtake[1,2,3], Sumi Hidaka[1,2], Takayuki Asahara[2] and Shuzo Kobayashi[1,2]

[1] Kidney Disease and Transplant Center, Shonan Kamakura General Hospital, 1-1370 Okamoto, Kamakura, Kanagawa, Japan, 2478533

[2] Shonan Research Institute of Innovative Medicine, Shonan Kamakura General Hospital, 1-1370 Okamoto, Kamakura, Kanagawa, Japan, 2478533

[3] Division of Regenerative Medicine, Department of Center for Clinical and Translational Science, Shonan Kamakura General Hospital, Kamakura, Okamoto 1-1370, Japan, 2478533

Supplementary Figures:

Supplementary Fig 1

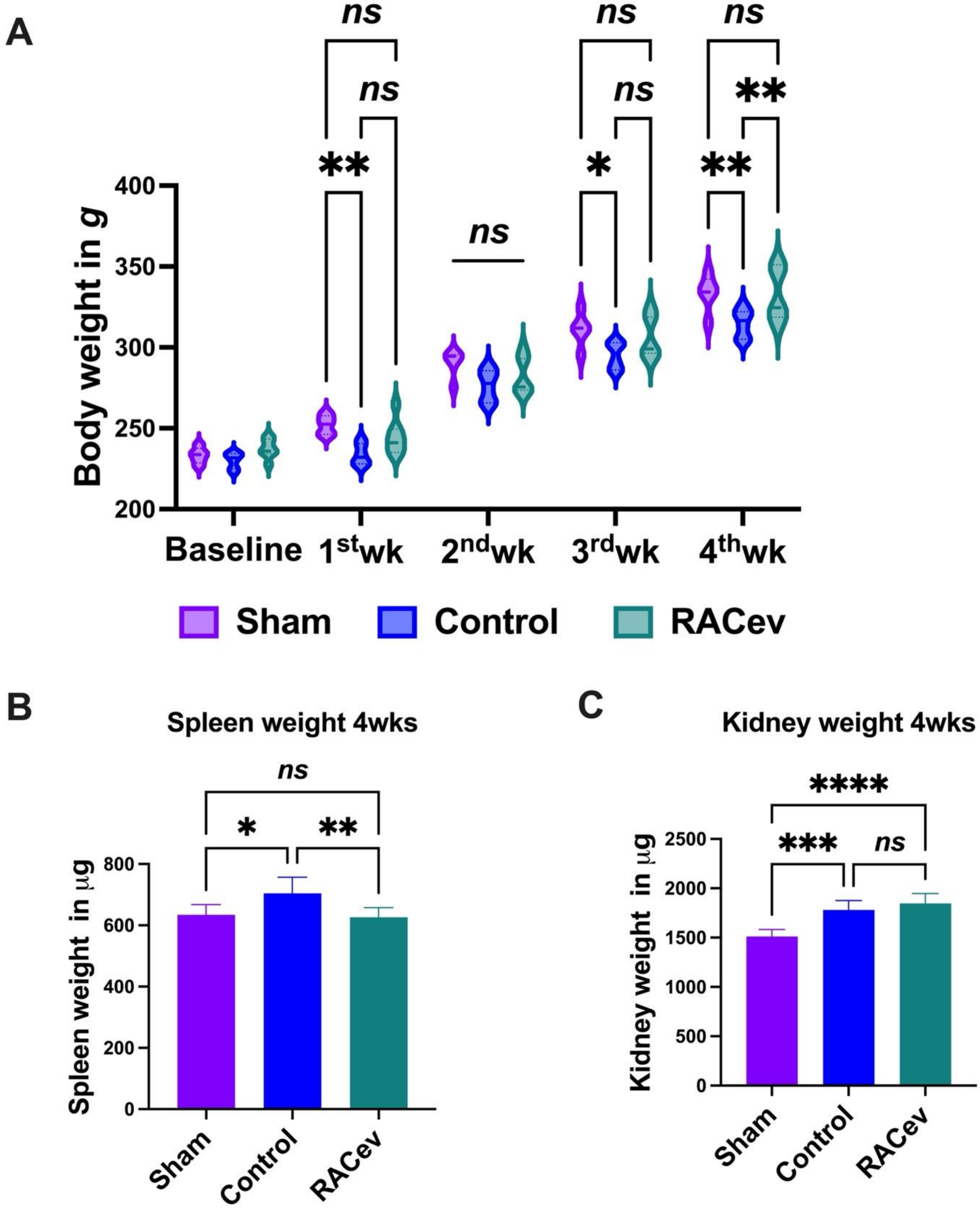

Supplementary Figure and Legend:

Fig S1. Animal body and organs weight at 4 weeks

(A) At four weeks, in the RACev transplanted group, body weight gain significantly increased than control group **(B)** Spleen weight at four weeks was enlarged in Control group while RACev was similar to Sham group. **(C)** Kidney gross weight at four weeks. * $P < 0.05$; ** $P < 0.01$; *** $P < 0.001$; **** $P < 0.0001$ for RACev *vs*. Control. RACev *vs*. Sham groups. Statistical significance was determined using a 2-way ANOVA for body weight and one-way for organs and followed by Tukey's multiple comparison test. The results are presented as mean ± SEM.
